# Habitat and Anthropogenic Determinants of Chinese Pangolin (*Manis pentadactyla*) Burrow Occupancy in Udayapur, Eastern Nepal: Implications for Site‐Specific Conservation

**DOI:** 10.1002/ece3.71493

**Published:** 2025-06-04

**Authors:** Bishal Bhandari, Bijaya Dhami, Nishan KC, Prakash Mahatara, Asmit Neupane, Shushma Gosai, Anush Udaya, Nikita Phuyal, Pramod Ghimire

**Affiliations:** ^1^ College of Natural Resource Management Agriculture and Forestry University Udayapur Nepal; ^2^ Department of Biological Sciences University of Alberta Edmonton Canada; ^3^ WWF Nepal Kathmandu Nepal; ^4^ Institute of Forestry Pokhara Campus Tribhuwan University Pokhara Nepal; ^5^ School of Forestry and Natural Resource Management, Institute of Forestry Tribhuvan University Kathmandu Nepal; ^6^ Faculty of Forestry Agriculture and Forestry University Hetauda Nepal

**Keywords:** anthropogenic disturbance, canopy cover, distribution, elevation, generalized linear mixed model

## Abstract

Chinese pangolins are found from lowlands to mid‐hills in Nepal and are increasingly vulnerable to extinction due to extensive illegal trade and habitat fragmentation, particularly outside the protected areas network. The information about their ecological preferences in human‐dominated landscapes beyond protected areas is essential for effective habitat management and conservation. This study aimed to assess the density and characteristics of Chinese pangolin burrows, and analyze the key ecological and anthropogenic factors influencing their burrow occurrence in Katari municipality, Udayapur District, Eastern Nepal. We employed a total of 52 strip transects, each of 500 m long and 20 m wide, divided into two 250‐m sections on either side of the main transect and spaced 200 m apart to examine the pangolin burrows. We recorded a total of 124 active burrows and 114 inactive burrows, with a density of 2.38 active burrows per hectare. A higher number of burrows (80.25%) were recorded in the forest habitat, suggesting a discrepancy in burrow distribution across the study area. The majority of the burrows were distributed at 601–800 m above sea level (33.61%), 35°–45° slope (53.78%), and 0%–25% canopy cover (52.94%). The Welch Two‐Sample *t*‐test suggested that there was a significant difference in the burrow opening diameter between feeding and resting burrows. Among the 15 pre‐determined ecological parameters measured during the study, a generalized linear mixed model (GLMM) identified 9 ecological parameters as significant variables influencing the Chinese pangolin burrow occurrence in the study area. These were elevation, habitat type, canopy cover, livestock grazing, anthropogenic disturbance, presence or absence of fire, and nearest distance to water sources, roads, and settlements. Long‐term habitat management plans and strategies are recommended, with an emphasis on minimizing anthropogenic disturbances both in forest and farmland areas, and proper preservation of nearby water sources.

## 1 Introduction

Anthropogenic pressures such as forest fires (Nepstad et al. 1999) and the use of chemical fertilizers in farmlands (Panta et al. 2023), along with activities like infrastructure development (Bhattarai et al. 2023), livestock grazing (Katuwal et al. 2017), and unsustainable forest harvesting have significantly contributed to the rapid decline of flora and fauna in recent years. These threats are more severe for threatened species (Pereira et al. 2004), particularly small mammals with a highly specialized ecological niche and less ecological flexibility (Büchi and Vuilleumier 2014; de Mattos de et al. 2021; Dhami, Neupane, et al. 2023). Therefore, the sustainable conservation efforts of globally threatened wildlife require science‐based information on how ecological and anthropogenic factors influence species' habitat use and occurrence, and such information can enable more targeted and site‐specific conservation strategies (Teixeira‐Santos et al. 2020; Searle et al. 2020; Nishan et al. 2023).

Pangolins belong to the mammalian order Pholidota, and they are one of the elusive mammals that are highly vulnerable to extinction due to habitat destruction and illegal trade (Wu et al. 2020; Phuyal et al. 2023). Among the nine extant species of pangolin found globally (Gu et al. 2023), two species, namely the Indian ( *Manis crassicaudata* Geoffroy, 1803) and Chinese ( *Manis pentadactyla* Linnaeus, 1758) pangolin, occur in Nepal (Baral and Shah 2008; Jnawali et al. 2011; DNPWC and DoF 2018). The Chinese pangolin is highly nocturnal and fossorial and frequently uses its powerful forelimbs to excavate burrows not only to search for ants or termites (i.e., feeding burrows) but also to create shelters for resting, giving birth, and nursing offspring (i.e., resting burrows) (Wu et al. 2020). Unlike feeding burrows, which are rarely revisited and degrade over time, resting burrows are permanent structures, frequently reused and maintained, and occasionally shared non‐simultaneously by different individuals (Wu et al. 2020; Sun et al. 2021). The species primarily feeds on ants and termites, playing a crucial ecological role in regulating their populations (Laundré and Reynolds 1993; Swart et al. 1999).

The extensive illegal trade of Chinese pangolin scales, driven by the increasing demand for their use in traditional Chinese medicine, has resulted in a significant population decline throughout its range, including Nepal (Heinrich et al. 2017; D'Cruze et al. 2018; Challender et al. 2020). The species is classified as ‘Critically Endangered’ on the International Union for Conservation of Nature (IUCN) Red List of Threatened Species (Challender et al. 2020) and is listed under Appendix I of the Convention on International Trade in Endangered Species of Wild Fauna and Flora (CITES 2020). In Nepal, the species is protected under the National Park and Wildlife Conservation (NPWC) Act, 1973 (GoN 1973).

The species occurs in a wide range of habitats, including primary and secondary tropical or subtropical forests, bamboo forests, grasslands, agricultural fields, and some degraded habitats (Zhang et al. 2022), with an elevation ranging from < 100 m to as high as 3000 m (Wu et al. 2020). In Nepal, the species is distributed across 25 districts up to an elevation range of about 2500 m above sea level (DNPWC and DoF 2018). The Chinese pangolin predominantly inhabits areas outside protected areas in Nepal, where human‐influenced activities, including livestock grazing, forest fires, and the collection of forest resources, are prevalent (DNPWC and DoF 2018; Sharma, Sharma, et al. 2020). They are distributed across the modified habitat, primarily within human‐dominated landscapes in proximity to villages, private forests, community forests, and cultivated land (Katuwal et al. 2017; Acharya et al. 2021; Suwal et al. 2020; Panta et al. 2023). This indicates that various habitat and anthropogenic variables could be influencing factors that govern the ecological requirements of the species to prefer such human‐dominated landscapes.

The conservation action plan for Chinese pangolins in Nepal suggests identifying the drivers of species occurrence and population dynamics (DNPWC and DoF 2018). However, the occurrence of pangolin species in Nepal, mostly outside protected areas, is not well documented, partly due to their globally characteristic low abundance and nocturnal behavior (Sharma, Sharma, et al. 2020; Dhami, Neupane, et al. 2023). In addition, scientific studies on the order Pholidota are limited in Nepal in comparison to the other mammalian orders (Bist et al. 2021). The occurrences of pangolins are affected by a range of ecological factors, including vegetation cover, water bodies, and soil type, alongside anthropogenic factors including land‐use changes and habitat fragmentation (Sharma, Rimal, et al. 2020; Dhami, Neupane, et al. 2023). Nationally, numerous scientific studies have focused on the habitat preference and distribution of pangolins, primarily within the mid‐hill region of Nepal, situated between a narrow elevation gradient of approximately 500 m above sea level (Thapa et al. 2014; Katuwal et al. 2017; Dhital et al. 2020; Acharya et al. 2021; Shrestha et al. 2021; Tamang et al. 2022; Dhami, Neupane, et al. 2023; Panta et al. 2023). However, there is very limited information on the habitat preference of the species, which covers a wide elevation range from Chure to the Mahabharat physiographic zone located outside a protected area in Nepal (Timsina and Baral 2020).

The National Pangolin Survey conducted in 2016 reported an unidentified species of pangolin in Udayapur district of Nepal (DNPWC and DoF 2018). Also, the faunal diversity survey in the Chure region of Nepal recorded Chinese pangolin in camera traps at Udayapur (Subedi et al. 2021). Despite some documented records of Chinese pangolins in Udayapur, comprehensive and appropriate information on their habitat and distribution remains deficient. Information regarding the habitat and distribution of species is often used as a baseline for devising conservation needs at a particular site. Detecting pangolins in the wild is challenging due to their elusive behavior and reliance on burrows. However, understanding the species' habitat preferences and the factors influencing habitat avoidance can provide valuable insights for planning effective habitat management strategies (Kellner and Swihart 2014; Aryal and Poudel 2018; Nishan et al. 2023; Dhami, Bhusal, et al. 2023). In this context, our study investigated burrow density and characteristics of the Chinese pangolin, as well as analyzed the key ecological and anthropogenic factors influencing burrow occurrence. Our findings provide baseline information for forest authorities and other conservation organizations to identify and prioritize key pangolin sites, emphasizing the development and implementation of effective habitat management and conservation strategies for both the short and long term.

## 2 Materials and Methods

### 2.1 Study Area

**FIGURE 1:**
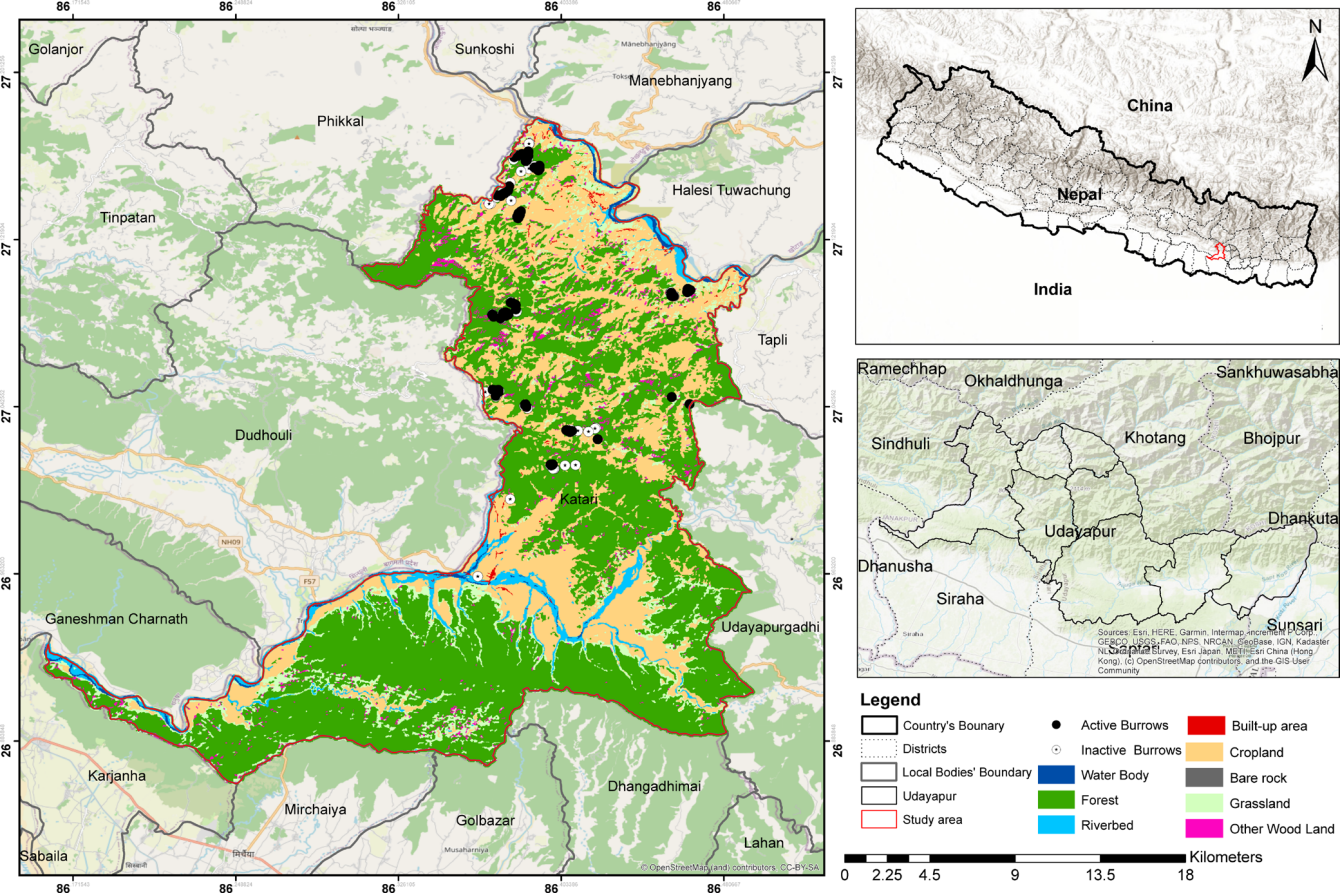
LULC map of the study area showing the distribution pattern of active and inactive burrows of Chinese pangolins. The source of the base layer of LULC is from the International Center for Integrated Mountain Development (ICIMOD), 2022.

The study was carried out in Katari Municipality in Udayapur District (Figure 1), Nepal. It is located in the eastern region of Nepal at approximately 26°57′47.3″N latitude and 86°22′15.4″E longitude, and ranges from 111 m to 1691 m above sea level. The municipality's total size is 424.89 km^2^. The study area is home to a variety of wildlife, including Chinese pangolin ( *Manis pentadactyla* ), Sloth bear ( *Melursus ursinus* Shaw, 1791), Striped hyena ( *Hyaena hyaena* Linnaeus, 1758), Indian crested porcupine ( *Hystrix indica* Kerr, 1792), Rhesus macaque ( *Macaca mulatta* Zimmermann, 1780), and Barking deer ( *Muntiacus muntjak* Zimmermann 1780) (Subba and Pokharel 2021). The study area is largely covered by private and community forests, with important floral species including Sal ( *Shorea robusta* ), Katus (*Castanopsis* spp.), Chilaune (*Schima wallichi*), and Khair (*Senegalia catechu*). Based on our field observations during the preliminary survey, community forests (CFs) and farmlands are found to be the prime pangolin habitat in the area. Therefore, seven CFs and farmland habitats were selected for this study.

### 2.2 Sampling Design and Data Collection

**FIGURE 2:**
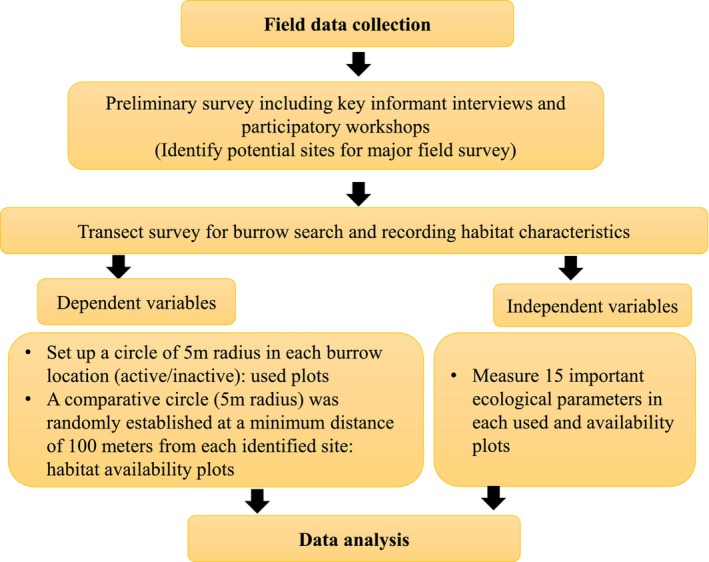
A conceptual framework for the study.

The entire survey included a preliminary survey, followed by a detailed habitat survey, conducted between April and May 2023 (Figure 2), assuming the activity of Chinese pangolins is high during their breeding season, i.e., February to July (Wu et al. 2020; Dhami, Neupane, et al. 2023).

#### 2.2.1 Preliminary Survey

The preliminary data collection process was conducted in two distinct phases to ensure thorough site selection for our study.

##### 2.2.1.1 Phase 1: Key Informant Interviews

The first phase involved conducting key informant interviews with division forest authorities, including the Divisional Forest Officer (DFO), rangers, and representatives from the Federation of Community Forestry Users Nepal (FECOFUN). These interviews were designed to gather detailed historical data on Chinese pangolin sightings, rescue operations, and habitat use. We informed each key informant about the objectives of our study and obtained their verbal consent before conducting the interview. Based on the information obtained, Katari Municipality was selected as the initial study site due to its documented records of Chinese pangolin activity, including multiple rescue events, and its significance as a habitat for the species. The study area was also selected based on logistical considerations such as accessibility.

##### 2.2.1.2 Phase 2: Participatory Workshop

In the second phase, we employed a participatory approach by organizing an informal workshop that included members from different Community Forest User Groups [CFUG] (*n* = 9), both men and women, along with DFO representatives, including rangers (*n* = 2). During the workshop, participants were provided with high‐resolution hard copy maps of the study site. They were tasked with identifying and demarcating locations where previous sightings of the species and its burrows occurred. This collaborative mapping exercise facilitated the integration of local ecological knowledge into our field survey. During the concluding phase of the workshop, a consensus was reached based on the data from the historical sighting incidents and rescue operations. This consensus allowed us to finalize the probable sites for the detailed field survey, ensuring that our research efforts were focused on the most relevant and significant areas, as identified through community input and expert consultation.

#### 2.2.2 Transect Survey and Recording of Habitat Characteristics

**FIGURE 3:**
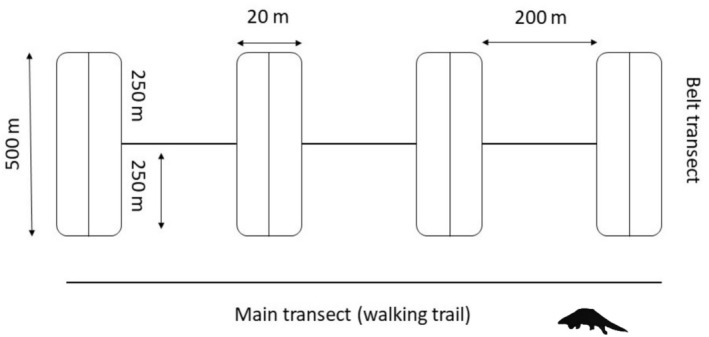
The layout of transects and their dimensions adopted during the study period.

Transects combined with the burrow count method are commonly used to study burrowing species such as pangolins, providing an effective approach for studying low‐density, rare, and elusive species (Ingram et al. 2019). We used curvilinear transects based on walking trails (human and wildlife) as the main transects, allowing the survey to follow the natural features of the habitat. While the natural trails may have inherent curvature and variability, we randomized the starting points of the transects within each habitat type to ensure even coverage and avoid bias toward any specific trail characteristics. 500 m long and 20 m wide transects were established, bisected by the main transect, with 250 m on the left and 250 m on the right. A minimum distance of 200 m was maintained between adjacent transects to avoid spatial overlap and reduce any potential bias caused by the natural trail paths (Figure 3).

A total of 52 transects were established, with 22 located in farmland and 30 in forest areas. Each transect was thoroughly searched for direct (active/inactive burrows) and indirect signs (digs, pug marks, scales, and scats) of pangolin presence. A team of two experienced field researchers, who had prior firsthand experience conducting pangolin surveys, carried out an intensive search within each belt transect to ensure that no burrows were missed. The transects were strategically chosen to represent all habitats proportionately based on their relative sizes. The identification of pangolin burrows was based on the characteristics outlined by DNPWC and DoF (2018) and Dhami, Neupane, et al. (2023). While the Chinese pangolin ( *Manis pentadactyla* ) and the Indian Crested Porcupine ( *Hystrix indica* ) may share habitats in the mid‐hills of Nepal (Jnawali et al. 2011), including our study area, there is a difference in their burrow characteristics. The Indian Crested Porcupine is known to excavate substantial burrows with strong front feet (Mukherjee et al. 2017), which may result in burrow structures that differ from those of the Chinese pangolin. Therefore, we identified burrows with a single entrance, having a semicircular shape, and featuring a heap of soil at the entrance, as Chinese pangolin burrows (DNPWC 2018). Active burrows were identified based on specific criteria, including the presence of fresh footprints at the burrow entrance, loose soil without dry fodder, and the absence of leaves or spider webs blocking the entrance. In contrast, burrows were classified as inactive if they displayed compact and dry soil, contained dry fodder, or had spider webs at the entrance. These classifications relied on observable physical characteristics, although environmental factors such as weather conditions were considered potential influences on these signs.

Each active burrow was further categorized as either a feeding or resting burrow based on distinctive features. Feeding burrows were characterized by their shallow and small structure with irregular entrances (Wu et al. 2003). The soil heaps around these burrows were smaller, consisting of fine and loose soil resulting from superficial digging activity, whereas the resting burrows featured more circular entrances and larger soil heaps that contained a mix of fine and coarse particles, reflecting the varied soil layers excavated during construction. The soil around resting burrows also appeared more compact due to repeated use and the need for structural stability (Sun et al. 2025). The opening diameter of each burrow was measured using a measuring tape, whereas the GPS location of the burrows was recorded using a handheld Garmin GPS Map 60 CSx.

At each pangolin burrow location (both active and inactive), a 5‐m radius circle centered on the burrow was established, following the method of Yahnke (2006), Bernard et al. (2014), and Panta et al. (2023). Additionally, for each of these circles, a comparable circle was set up at least 100 m away in a randomly chosen direction, following Neupane et al. (2022) and Dhami, Bhusal, et al. (2023). These comparative plots represented average habitat conditions, independent of pangolin presence. Consequently, some of these comparative circles also contained burrows. All circles with burrows were classified as “used plots,” while those without burrows were categorized as “habitat availability plots.” In all these used and availability plots, we recorded 15 ecological parameters, including elevation, habitat type, aspect, slope, canopy cover, presence or absence of ant and termite colonies, presence or absence of fire, livestock grazing, anthropogenic disturbance (logging, firewood collections, agricultural practices and tourism related construction), soil color, soil type, soil pH, and the nearest distances to water resources, roads, and settlements (Panta et al. 2023; Dhami, Neupane, et al. 2023). While proximity to roads and settlements are indeed recognized as anthropogenic disturbances, we chose to analyze them separately as continuous variables (Dhami, Neupane, et al. 2023). By treating distance to roads and settlements as continuous variables, we aim to capture these gradient effects more precisely, providing a comprehensive understanding of how different anthropogenic disturbances influence pangolin ecology.

The canopy cover was recorded using the mobile software called “GLAMA application” (Tichý 2016), elevation using GPS (Garmin GPS Map 60 CSx), slope, and aspect using the Santo clinometer. We recorded the presence or absence of livestock grazing, anthropogenic disturbance, presence or absence of fire, and ant or termite colonies through direct field observation during the survey. The road and settlement shape files were extracted from the open street map, and the nearest distance from the species burrow location was calculated using the near analysis tool in ArcGIS10.8. The distance to the nearest water source was calculated by the near analysis tool in ArcGIS by extracting the shape files of the water surface from the digital elevation model (DEM) and Landsat image. Firstly, the shape file of the water source was extracted from a DEM (12.5 m resolution) and from Landsat Image 8 (USGS 2021). Then, the nearest distance to water resources was calculated using the “Nearer function” in ArcGIS 10.8 with the presence locations (geographic coordinates) of the Chinese pangolin burrows. Moreover, we collected soil samples (approximately 100–150 g) from each plot and labeled them in zip‐lock bags for further laboratory analysis. Soil color was analyzed using Munsell's color chart, soil pH was calculated using a pH measuring instrument (Lutron BPH‐231), and the soil texture was analyzed using the hydrometer method (Table 1). We categorized pangolin habitat into two different types, which included forest and farmland, as per the burrow occurrence. Grassland and shrub land habitats were not considered due to the very few availabilities of burrows in our study area.

**TABLE 1:** Method adopted for testing different soil parameters in soil laboratory.

Soil parameters	Quantity	Instruments/methods used
Soil pH	10 g	Lutron BPH‐231
Soil Color	> 5 g	Munsell Color Chart
Soil Texture	50 g	Hydrometer Method (Bouyoucos 1962)

### 2.3 Data Analysis

Based on the collected GPS location of burrow presence of species, a distribution map was prepared using ArcGIS 10.8 (Figure 1). The burrow distribution pattern of the study area was determined by calculating the ratio of variance and mean (*S* ^2^/*a*) (Odum 1971).

$$\text{Variance}=S^{2}=1/n\;\Sigma \;{\left(x-a\right)}^{2}$$

where, *x*, sample value; *a*, mean value; If *S* ^2^/*a* = 1, Distribution is random; If *S* ^2^/*a* < 1, Distribution is uniform; If *S* ^2^/*a* > 1, Distribution is clumped.

The frequency of burrows associated with the occurrence of each ecological parameter (P_0_) was calculated (Panta et al. 2023; Wu et al. 2003).

$$\left(P_{0}\right)=\left(\text{Frequency of burrow occurrence of}\;a\;\text{specific habitat feature}/\text{Total number of burrows observed}\right)\times 100$$

Similarly, the Welch two‐sample *t*‐test was used at the 5% significance level to analyze the statistical difference in opening burrow diameter between resting and feeding burrows.

#### 2.3.1 Factors Influencing the Occurrence of Chinese Pangolin Burrows

A generalized linear mixed model (GLMM) was used to examine the factors influencing the burrow selection by Chinese pangolins in the study area. The analysis was conducted using the “glmmTMB” package (Brooks et al. 2017). The presence or absence of pangolin burrows (coded as 1 for presence and 0 for absence) at each sampled location served as the dependent variable. As independent variables, we included fifteen ecological parameters, namely elevation, habitat type, aspect, slope, canopy cover, presence or absence of ant and termite colonies, presence or absence of fire, livestock grazing, anthropogenic disturbance, soil color, soil type, soil pH, and the nearest distances to water sources, roads, and settlements. To account for site‐specific variability, we included wards (the smallest administrative unit in Nepal) as a random effect. Prior to modeling, we log‐transformed the nearest distance to roads and settlements. These transformations help improve the assumption of normally distributed residuals and reduce heteroscedasticity. We also assessed collinearity among the predictors using Pearson's correlation coefficient, and no variable pair exhibited a correlation coefficient exceeding the threshold of 0.6. Given these results, we proceeded with detailed model construction. To identify the most appropriate model, we employed a model selection procedure based on Akaike Information Criterion (AIC) values. The “MuMIn” package (Barton and Barton 2015) was used to perform model dredging, generating and comparing all possible subsets of the predictor variables. The model with the lowest AIC value was selected as the best‐fitting model, ensuring an optimal balance between model complexity and goodness‐of‐fit at 95% confidence intervals. Finally, we assessed the assumptions and overall fit of the selected model using the “DHARMa” package (Hartig 2019). Diagnostic checks were performed on residual patterns and over‐dispersion to validate the robustness of the model's inferences. All the analyses including Welch two‐sample *t*‐test and generalized linear mixed model were computed using R × 64 4.0.3″ (http://cran.r‐project.org/) (R Core Team 2020).

## 3 Results

### 3.1 Habitat Distribution and Burrow Characteristics

A total of 238 Chinese pangolin burrows were recorded in the study area, with 191 in forest and 47 in farmland habitats. Out of these, 124 were classified as active and 114 as inactive. The overall distribution of Chinese pangolin burrows in the study area was clumped, as the observed variance to mean ratio (2.188) is > 1. The overall active burrow density in the study area was 2.38 burrows per hectare, with higher densities observed in forest habitats compared to farmland. Considerable variation in burrow density was observed across the seven community forests, with the highest density in Soluvir Vangwari CF and the lowest in Patnaharit CF (see Table 2 for detailed values).

**TABLE 2:** Burrow density of Chinese pangolin in different community forests of study area.

Community forest	Active burrow	Inactive burrow	Total burrow	Active burrow density/ha	Total burrow density/ha
Soluvir Vangwari	35	20	55	5.84	9.17
Sunkoshi	10	8	18	3.33	6
Chulitham	17	11	28	3.4	5.6
Gurdhum dada	22	14	36	5.5	9
Bisauna	15	15	30	3	6
Chisapani	5	11	16	1.25	4
Patnaharit	2	6	8	0.67	2.67
Overall	106	85	191	3.53	6.37

**FIGURE 4:**
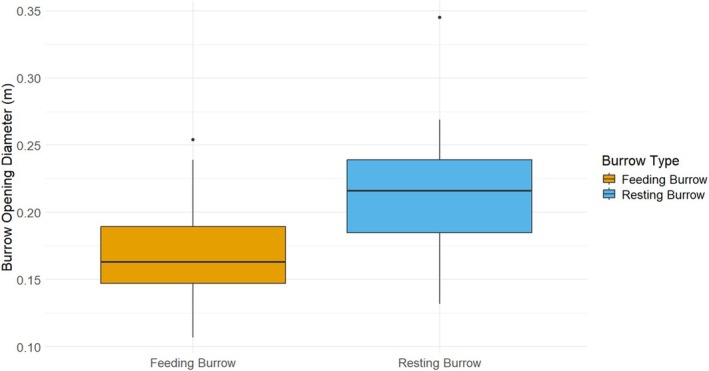
Box plot showing the comparison of opening diameters between resting and feeding burrows.

The average burrow opening diameter of feeding burrows was 0.17 ± 0.03 m (*n* = 95), and the average burrow opening diameter of resting burrows was 0.21 ± 0.04 m (*n* = 29) (Figure 4). The results indicate a statistically significant difference in burrow opening diameters between feeding and resting burrows (Welch Two‐Sample *t*‐test: *t* = −4.9734, df = 35.955, *p* < 0.001). The negative *t*‐value suggests that the burrow opening diameter is notably smaller in the feeding burrows compared to the resting burrows.

### 3.2 Ecological Parameters Associated With the Observed Burrows

Most of the burrows were recorded in forest habitats within an elevation range of 601 to 800 m above sea level, in areas with a north‐east and north‐west facing aspect, with less canopy cover, steep terrain, and in brown soil having a neutral pH with silt texture. Most of the burrows were distributed in the vicinity of water sources, human settlements, and roads. Furthermore, the majority of burrows occurred in areas with less anthropogenic disturbance and no evidence of fire. Moreover, comparatively more burrows were observed in areas less impacted by livestock grazing and in areas without the presence of ants or termite colonies (see Table 3).

**TABLE 3:** Occurrence frequency of observed burrows of Chinese pangolin with respect to different ecological parameters.

Ecological parameters	Sub‐category	Total burrows	*P*_o_ (%)
Habitat	Forest	191	80.25
	Farmland	47	19.75
Elevation (m)	400–600	33	13.87
	601–800	80	33.61
	801–1000	36	15.13
	1001–1200	35	14.71
	1201–1400	18	7.56
	1401–1600	36	15.13
Aspect	E	4	1.68
	W	17	7.14
	N	16	6.72
	S	16	6.72
	NE	47	19.75
	NW	47	19.75
	SE	46	19.33
	SW	45	18.91
Slope (degree)	0–15	4	1.68
	15–25	24	10.08
	25–35	82	34.45
	35–45	128	53.78
Canopy cover (%)	0–25	126	52.94
	26–50	47	19.75
	51–75	42	17.65
	> 75	23	9.66
Distance to nearby road (m)	0–100	116	48.74
	100–200	65	27.31
	200–300	42	17.65
	300–400	15	6.30
Distance to nearest water resources (m)	0–1000	174	73.11
	1000–2000	58	24.37
	> 2000	6	2.52
Distance to nearest human settlement (m)	0–1000	29	12.18
	1000–2000	82	34.45
	2000–3000	52	21.85
	3000–4000	69	28.99
	4000–5000	3	1.26
	> 5000	3	1.26
Presence or absence of ants/termites	Presence	104	43.70
	Absence	134	56.30
Livestock grazing	Presence	102	42.86
	Absence	136	57.14
Anthropogenic disturbance	Yes	61	25.63
	No	177	74.37
Presence or absence of fire	Yes	88	36.97
	No	150	63.03
Soil texture	Sandy	74	31.09
	Silt	88	36.97
	Clay	76	31.93
Soil pH	< 4.5	3	1.26
	4.5–5.5	26	10.92
	5.5–6.5	66	27.73
	6.5–7.5	91	38.24
	> 7.5	52	21.85
Soil color	Black	9	3.78
	Brown	41	17.23
	Dark brown	5	2.10
	Dark greenish gray	12	5.04
	Dark gray	11	4.62
	Dark reddish brown	16	6.72
	Dark reddish gray	12	5.04
	Dark yellowish brown	10	4.20
	Light brown	34	14.29
	Gray	4	1.68
	Light reddish brown	20	8.40
	Pale red	9	3.78
	Pinkish gray	24	10.08
	Red	3	1.26
	Reddish brown	4	1.68
	Weak red	7	2.94
	Yellowish red	17	7.14

### 3.3 Ecological Variables Influencing the Occurrence Probability of Chinese Pangolin Burrows

**FIGURE 5:**
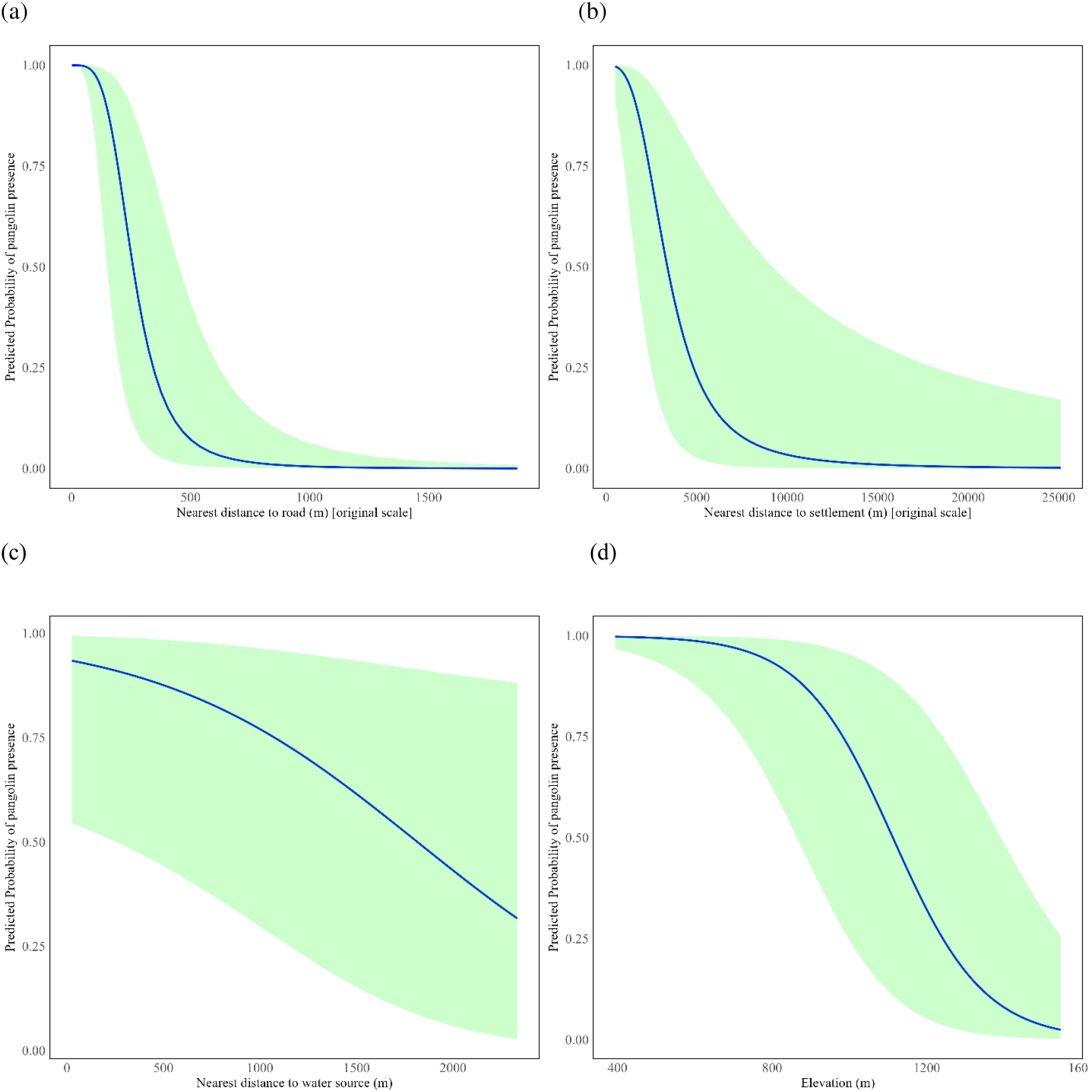
The negative relationship between the probability of burrow occurrence of Chinese pangolin with respect to (a) nearest distance to road, (b) nearest distance to settlement, (c) nearest distance to water sources, and (d) elevation.

The model coefficients from our analysis showed that elevation, habitat type, canopy cover, livestock grazing, anthropogenic disturbance, presence or absence of fire, and nearest distances to roads, settlements, and water sources significantly influenced the occurrence probability of Chinese pangolin burrows (Table 4). The probability of burrow occurrence increased in forest habitats and areas with lower canopy cover (0%–25%). In contrast, it decreased with higher elevation, the presence of anthropogenic disturbance, livestock grazing, and fire, as well as with increasing distance from roads, settlements, and water sources (Figure 5). The model demonstrated a good fit, with no evidence of non‐random patterns in the residuals, as indicated by diagnostic tests in the DHARMa package (KS test: *p* = 0.99; dispersion test: *p* = 0.86; outlier test: *p* = 0.7) (Figure S1).

**TABLE 4:** Model averaged coefficients influencing the probability of occurrence of Chinese pangolin burrows in the study area, surveyed during February– July 2023.

Predictor	Estimate	SE	*z* value	Pr(>|z|)
(Intercept)	58.73414	10.86770	5.404	6.50e−08***
Habitat Type: Forest	3.31934	0.882322	3.762	0.000169***
Elevation	−0.00850	0.001493	−5.692	1.25e−08***
Canopy Cover: 0–25	2.68600	0.959833	2.798	0.005136**
Canopy Cover: 26–50	−0.54108	0.879187	−0.615	0.538273
Canopy Cover: 51–75	−0.74871	0.921463	−0.813	0.416491
Log Nearest Distance to Roads	−3.87190	0.522591	−7.409	1.27e−13***
Log Nearest Distance to Settlements	−3.08117	0.921210	−3.345	0.000824***
Distance to nearest water sources	−0.00149	0.000709	−2.096	0.036126*
Livestock Grazing: Yes	−1.35903	0.633530	−2.145	0.031939*
Anthropogenic disturbance: Yes	−4.08960	0.756651	−5.405	6.49e−08***
Presence or Absence of Fire: Yes	−1.13853	0.538898	−2.113	0.034626*
Soil pH	−0.67997	0.431484	−1.576	0.115054

*Note:* Significance codes: *p < 0.05, **p < 0.01, ***P < 0.001.

## 4 Discussion

The distribution of Chinese pangolin burrows within our study area was found to be non‐uniform or clumped. A similar non‐uniform distribution was also observed by Dhami, Neupane, et al. (2023), Panta et al. (2023), Suwal (2011), and Bhandari and Chalise (2014). The reason behind the non‐uniformly distributed Chinese pangolin burrows in our study area may be due to the availability of prey species, favorable vegetation, and habitat conditions. Within our study area, the active burrow density of the Chinese pangolin was 2.38 burrows per hectare. In comparison, Suwal (2011) recorded 8 burrows per hectare, while Dhami, Neupane, et al. (2023) recorded a total burrow density of 1.04 per hectare. Also, we found a greater number of burrows in the forest habitat compared to the farmlands. This may be attributed to the fact that lower anthropogenic disturbance was observed in the forest habitat, which might reduce the chances of direct human interaction, potentially creating a suitable habitat for pangolins.

Our study revealed that the average burrow opening diameter for feeding burrows was smaller than that of resting burrows. This suggests that pangolins prefer to make burrows with a narrower diameter during foraging to save time and energy while constructing the burrow. This is also advantageous for defense during an attack from predators (Hua et al. 2020). In contrast, resting burrows are deeper and larger for protection against the environmental conditions, providing security, insulation, and ample space for the pangolin to maneuver and adjust its position during periods of inactivity or rest (Karawita et al. 2018). Moreover, multiple borrowings by one or multiple individuals may account for the larger burrow opening diameter of resting burrows.

The forest habitat was found to be a statistically significant factor influencing the probability of Chinese pangolin burrow occurrence in our study area, which is similar to the findings of Suwal et al. (2020). Forest vegetation in our study area was dominated by Sal ( *Shorea robusta* ) and Chir Pine (*Pinus roxburghii*). However, Chinese pangolins occur in various types of habitats, such as meadows, agricultural fields, broadleaf and coniferous forests, bamboo forests, limestone forests, primary and secondary tropical forests, and grasslands (Gurung 1996; Azhar et al. 2013; Katuwal et al. 2015). The occurrence of a higher number of burrows in forest areas may be due to the presence of tree stumps and snags in the forest area, providing a good nesting area for ants and termites and potentially serving a higher prey abundance for pangolins (Dorji et al. 2020).

Elevation was also found to have an inverse relationship with the probability of occurrence of Chinese pangolin burrows in our study area. We observed burrows between the elevation range of 400–1600 m above sea level, with the highest number of burrows at an elevation of 601–800 m above sea level. In line with our findings, Dorji et al. (2020) also revealed an inverse relationship between elevation and the number of burrows in the Samtse district in Southwestern Bhutan. Elevation could potentially shape the distribution of Chinese pangolin burrows, as the availability of termites and ants can decrease with an increase in elevation, which can contribute to a decline in the burrow's density (Swart et al. 1999; Hemachandra et al. 2014). Additionally, changes in climatic factors such as temperature, solar radiation, and precipitation along the elevation gradient could possibly result in a declining number of burrows (Elith and Leathwick 2009).

Our study showed that the probability of Chinese pangolin burrow occurrence significantly increases in the areas with open canopy cover (0%–25%). Other studies from the mid‐hills region of Nepal (Bhandari and Chalise 2014; Dhital et al. 2020; Acharya et al. 2021) have also found a higher prevalence of pangolin burrows in areas of low canopy cover, which supports our findings. Such preference might be influenced by prey availability as these low canopy cover areas boast higher densities of ants and termites due to the large number of fallen logs and cut stumps, which was also observed in our study area (Richer et al. 1997; Dorji et al. 2020). In contrast, denser canopy cover might alter microclimatic conditions, such as soil temperature and moisture, affecting prey availability (Cornelius and Osbrink 2010; Axelsson and Andersson 2012). However, this contradicts the findings of Katuwal et al. (2017), Suwal et al. (2020), and Shrestha et al. (2021), who reported that pangolins prefer dense canopy cover.

Our analysis revealed a negative influence of anthropogenic disturbances on the probability of Chinese pangolin burrow occurrence. Anthropogenic disturbances, such as the collection of firewood and fallen logs, as well as the use of chemical fertilizers, primarily urea and diammonium phosphate (DAP) in farmlands, were observed in the study area. Such use of chemical fertilizers affects the abundance and diversity of soil‐litter arthropods, including ants (Nsengimana et al. 2023), ultimately decreasing the probability of detecting pangolin burrows (Katuwal et al. 2017; Sharma, Rimal, et al. 2020; Bhattarai et al. 2023). Furthermore, Shannon et al. (2016) revealed that anthropogenic noise and other disturbances significantly impact the ecology and behavior of wildlife, including Chinese pangolins, which prefer to inhabit wild and remote areas with minimal disturbance.

The study also found livestock grazing as a significant factor affecting the occurrence of Chinese pangolin burrows, as they are less likely to prefer the habitat with livestock grazing pressure. Similar findings were also reported by Sharma, Rimal, et al. (2020) and Katuwal et al. (2017) in their studies. This could be because our study area covers CFs and farmland areas, which are frequently near human settlements and are more susceptible to overgrazing and livestock pressure. Such excessive grazing, especially by large hoofed cattle (such as cows, oxen, and buffalo observed in our study area), may lead to the blockage and destruction of ant and termite mounds, as well as Chinese pangolin burrows. They therefore tend to avoid such habitats (Katuwal et al. 2017). Additionally, overgrazing changes the microclimate of the forest understory (Belsky et al. 1993; Yong‐Zhong et al. 2005) by reducing soil moisture and understory vegetation, which can decrease habitat suitability for detritivores (Bromham et al. 1999) and potentially impact the prey base of Chinese pangolins.

From our study, it was revealed that the presence of fire negatively affects the occurrence of Chinese pangolin burrows within the study area. The research was conducted during April and May, which is the dry season in Nepal. During the period, particularly from March to May, it is characterized by a significant increase in forest fires, especially in the mid‐hills region, including our study area (Mishra et al. 2023), which drastically reduces vegetation cover by consuming underbrush in forest habitats. These underbrushes, which include shrubs, fallen logs, and leaf litter, are essential components of the pangolin habitat as they harbor abundant ants and termites. Frequent fires, however, deplete these food sources and reduce habitat suitability for pangolins (Richer et al. 1997; Katuwal et al. 2017). Additionally, controlled burning practices, commonly employed by CFs within our study area to promote forest regeneration, may further disrupt pangolin habitats.

Our analysis revealed a significant association between the probability of burrow occurrence and distance to the nearest water source. The probability of Chinese pangolin burrows occurring increases in areas close to water sources. In line with our findings, Shrestha et al. (2021) also revealed that Chinese pangolin burrows were found within 150 m of the nearest water source. Similarly, Dorji et al. (2020) reported a higher number of burrows near water sources. Chinese pangolins intake water frequently (Suwal 2011) and the presence of burrows near the water source could aid in conserving vital energy (Shrestha et al. 2021). Furthermore, ant and termite populations are abundant in moist places near water sources, contributing to a preferable habitat for Chinese pangolins (Cornelius and Osbrink 2010; Dhami, Neupane, et al. 2023).

The findings show that Chinese pangolins prefer to construct burrows closer to human settlements. We observed most of the burrows in the community forest habitat near human settlements, where ants and termites are abundant. This is consistent with the findings of Sharma, Sharma, et al. (2020), which reported 51% of occurrence plots within 1000 m of settlements. The proximity of pangolin burrows to settlements may reflect the dispersed nature of settlements within forested areas and the availability of resources. Conversely, Katuwal et al. (2017) found higher pangolin occurrences in less disturbed areas (> 1000 m from settlements). This discrepancy highlights the influence of varying land‐use practices on habitat selection. In our study area, we observed many unproductive farmlands near human settlements. This might be due to the increasing migration of people from our study area to nearby cities or lowland areas. Thus, the observed burrow occurrence patterns may be further influenced by shifts in land use, such as the conversion of farmlands to forested areas in proximity to human settlements.

As a part of the community forest program, many community forest user groups have constructed road channels within many forest blocks to facilitate the transportation of forest‐based resources, and such channels also act as fire lines, preventing forest fires from spreading in the dry season. As a result, we observed a higher number of burrows near roads, potentially increasing their risk of exposure to human interactions and illegal activities, including hunting and poaching (Katuwal et al. 2017). Our study revealed an inverse relationship between the probabilities of Chinese pangolin burrow occurrence and the nearest distance from the road in the study area. In contrast to our findings, Dhami, Neupane, et al. (2023) found that the probability of finding a burrow gradually decreases with an increase in the distance from the nearest road. However, Katuwal et al. (2017) observed more burrows near busy walking trails, with the highest number within 50 m of these trails, which also supports our findings.

## 5 Conclusion and Conservation Implications

This study provides comprehensive insights into the distribution of Chinese pangolin burrows and the ecological factors influencing their burrow site selection in Katari Municipality, Udayapur District, Eastern Nepal. Most burrows were recorded in the forest habitat, with a higher proportion observed at elevations ranging from 601 to 800 m above sea level. We found a significant difference in the diameter of burrow openings between feeding and resting burrows. Habitat and anthropogenic factors, including habitat type, elevation, canopy cover, anthropogenic disturbance, presence or absence of fire, livestock grazing, nearest distance to roads, water sources, and settlements, were found to have a significant influence on the occurrence of Chinese pangolin burrows. The occurrence of burrows was positively associated with forest habitats and areas with lower canopy cover, while it was negatively associated with higher elevation, greater distances from roads, settlements, and water resources, as well as the presence of anthropogenic disturbances, livestock grazing, and fire.

We recommend that forest authorities and conservation organizations implement site‐specific habitat management measures for pangolin conservation, with a focus on minimizing anthropogenic disturbances both in forest and farmland areas. Available water sources in areas where pangolins are found should be properly managed and conserved. Furthermore, we also suggest practicing a community‐based pangolin conservation model to promote community engagement, ultimately minimizing prevalent anthropogenic threats within our study area. We acknowledge that our measures of indirect presence of pangolins (i.e., burrow observations) have their limitations in precisely estimating the occurrence probability of burrows. Future studies should integrate camera trapping along with burrow observation data, as well as various bio‐climatic variables to ensure more science‐driven information regarding pangolin ecology and habitat occupancy.

## Author Contributions

**Bishal Bhandari:** conceptualization (lead), formal analysis (equal), funding acquisition (lead), investigation (lead), methodology (equal), writing – original draft (equal). **Bijaya Dhami:** conceptualization (equal), formal analysis (lead), supervision (equal), writing – original draft (equal). **Nishan KC:** conceptualization (equal), formal analysis (equal), writing – review and editing (equal). **Prakash Mahatara:** investigation (equal), writing – original draft (equal). **Asmit Neupane:** writing – review and editing (equal). **Shushma Gosai:** data curation (equal), investigation (equal). **Anush Udaya:** writing – review and editing (equal). **Nikita Phuyal:** writing – review and editing (equal). **Pramod Ghimire:** supervision (equal), writing – review and editing (equal).

## Ethics Statement

All the necessary ethical considerations of the study were approved by the Research Committee of Agriculture and Forestry University, Nepal.

## Conflicts of Interest

The authors declare no conflicts of interest.

## Supporting information


Data S1.

Data S2.

Figure S1.

## Data Availability

We have uploaded the data as Data S1 and S2 for reviewers and publication.
