# Supplementary material for: Habitat and Anthropogenic Determinants of Chinese Pangolin (Manis pentadactyla) Burrow Occupancy in Udayapur, Eastern Nepal: Implications for Site‐Specific Conservation
Source: Ecol Evol. 2025 Jun 4;15(6):e71493. doi: 10.1002/ece3.71493 (PMC12134491; doi:10.1002/ece3.71493)
Supplement: Supplementary file 3 — Figure S1. [file ECE3-15-e71493-s003.docx]

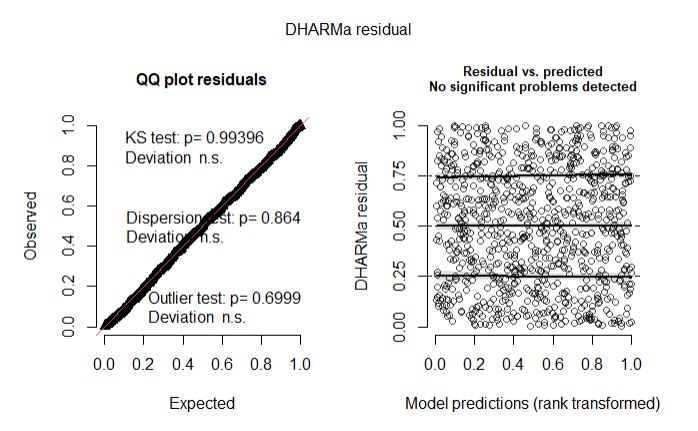


**Supplementary Figure S1.** Model diagnostic plots assessing residual patterns and over dispersion using the DHARMa package. (Left) Quantile-quantile (QQ) plot of residuals showing no significant deviation from expected values (KS test: p = 0.99396). (Right) Residuals vs. predicted values plot, indicating no significant patterns or over dispersion (dispersion test: p = 0.864, outlier test: p = 0.6999). These results confirm that the model provides a good fit with no major violations of distributional assumptions.
